# Investigating the Effects of PA66 Electrospun Nanofibers Layered within an Adhesive Composite Joint Fabricated under Autoclave Curing

**DOI:** 10.1021/acsomega.3c03419

**Published:** 2023-08-29

**Authors:** Gözde Esenoğlu, Metin Tanoğlu, Murat Barisik, Hande İplikçi, Melisa Yeke, Kaan Nuhoğlu, Ceren Türkdoğan, Seçkin Martin, Engin Aktaş, Serkan Dehneliler, Ahmet Ayberk Gürbüz, Mehmet Erdem İriş

**Affiliations:** † Department of Mechanical Engineering, Izmir Institute of Technology, Urla 35340, Izmir , Turkey; ‡ TUSAŞ (Turkish Aerospace Industries Inc.), Kahramankazan, 06980 Ankara, Turkey; § Department of Mechanical Engineering, University of Tennessee at Chattanooga, Chattanooga, Tennessee 37403, United States; ∥ Department of Civil Engineering, Izmir Institute of Technology, Urla 35340, Izmir , Turkey

## Abstract

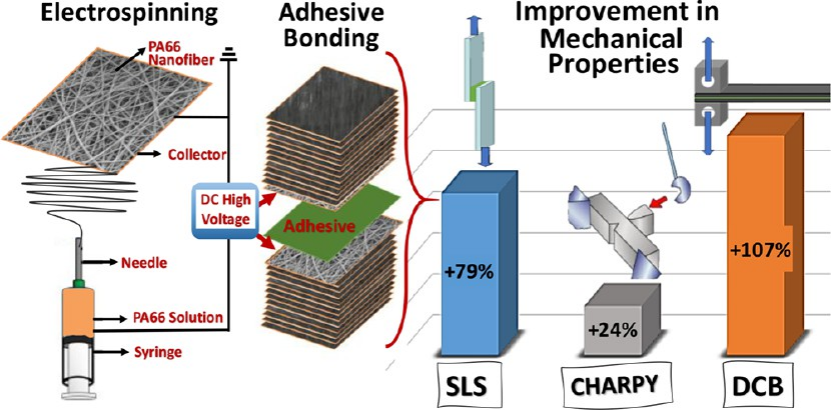

Enhancing the performance of adhesively joined composite components is crucial for various industrial applications. In this study, polyamide 66 (PA66) nanofibers produced by electrospinning were coated on unidirectional carbon/epoxy prepregs to increase the bond strength of the composites. Carbon/epoxy prepregs with/without PA66 nanofiber coating on the bonding region were fabricated using the autoclave, which is often used in the aerospace industry. The single lap shear Charpy impact energy and Mode-I fracture toughness tests were employed to examine the effects of PA66 nanofibers on the mechanical properties of the joint region. Scanning electron microscopy (SEM) was used to investigate the nanofiber morphology and fracture modes. The thermal characteristics of Polyamide 66 nanofibers were explored by using differential scanning calorimetry (DSC). We observed that the electrospun PA66 nanofiber coating on the prepreg surfaces substantially improves the joint strength. Results revealed that the single lap shear and Charpy impact strength values of the composite joint are increased by about 79 and 24%, respectively, by coating PA66 nanofibers onto the joining region. The results also showed that by coating PA66 nanofibers, the Mode-I fracture toughness value was improved by about 107% while the glass transition temperature remained constant.

## 1 Introduction

Composite materials are materials with higher strength-to-weight ratio characteristics. For this reason, the use of composites continues to increase, especially in the aviation industry.^1−3^ However, joining these composite components is still challenging, as it is considered the weakest link within a composite structure. The performance of joints is influenced by various factors, including environmental conditions. Such as type of adhesive, surface treatment, temperature, load, and humidity.^4−8^ Traditionally, two established methods exist to join components, i.e., mechanical fastening and adhesive bonding. Mechanical connections, achieved through bolts or rivets that involve drilling, can lead to problems in composite materials. When composite materials are drilled, delamination, fiber structure degradation, and stress concentrations can occur around the holes. To address these concerns, adhesively bonded joints have gained popularity due to their inherent advantages over mechanical fastening. The use of adhesively bonded joints results in a more uniform load transfer across a larger area, eliminating the need for fastening holes and fasteners. This, in turn, reduces stress concentrations and weight gain in the overall structure. Moreover, in these applications, the adhesion properties of the adhesive are strengthened by applying surface treatments to the bonded material. In the literature, the strength of the joining processes is evaluated by the lap shear test. Researchers have identified three primary methods of adhesive joining for manufacturing composite structures.^9^ The first method involves a simultaneous joining and curing process of both parts, known as cocuring. This can be carried out with or without the use of an adhesive, as both parts are uncured during the process. The second method is cobonding, where a cured part is attached to an uncured part using an adhesive on the joining surface. The third method is secondary bonding, where two cured same or different parts are joined by applying an adhesive to the joining surfaces. According to the majority of researchers, the strongest bond strength performance is typically attained when employing cocuring or secondary bonding methods.^8^ Particularly when joining complex structures, secondary bonding has demonstrated superior performance compared to the other two methods, as indicated in previous studies.^9−11^ In-depth investigations by researchers have focused on understanding failure mechanisms occurring at the joint interfaces. Adhesive failure, caused by the breakdown of the bond between the adhesive and the structure, is one of the common problems encountered.^9,12^ Interestingly, studies have indicated that increasing the adhesive thickness contributes to better adhesion of the adhesive to the surface.^8,13^

Recently, there has been increasing interest in the use of an electrospinning method to improve the mechanical and chemical properties of materials.^14−19^ Researchers have actively explored the use of nanofibers as an interface component during the fabrication of composite materials from prepregs, with the aim of enhancing material strength. Electrospinning is the most effective method of producing nanofibers from polymer materials. Nanofiber diameters can be precisely tuned by changing the parameters used in this method (solution type, voltage, environmental factors, etc.). This level of control over the electrospinning process contributes to the versatility and tailorability of nanofibers for various composite applications.^20,21^ Extensive research has been conducted on electrospun nanofibers due to their remarkable reinforcing capabilities in polymer composites. This is primarily attributed to their ultrafine size, exhibiting high mechanical strength, and possessing an exceptionally large surface area. The unique combination of these properties makes electrospun nanofibers promising candidates for enhancing the performance of polymer composites in various applications. Nanofibers produced by electrospinning can be prepared from various polymer solutions and by incorporation of various fillers. It was shown by multiple studies that the addition of nanofibers to composite structures enhances the mechanical properties.^22^ Recent studies have introduced the utilization of thermoplastic nanofibers as a means to enhance composite strength while simultaneously preserving the in-plane mechanical properties. There are many studies dedicated to exploring the mechanical performance of different materials. For example, adding poly e-caprolactone nanofibers produced by electro-spinning was found to increase the fracture toughness of composite materials.^23^ Similarly, adding polyacrylonitrile (PAN) nanofibers between carbon fabrics was shown to increase the load capacity of pin-bonded composite laminates.^24^ Bilge et al. showed that incorporating P(St-*co*-GMA) nanofibers into composite structures yields an 18% increase in the tensile strength.^25^ Moreover, the research demonstrated that by adding nanofiber interlayers, comprising 9 wt % of the composite, to regions exposed to high stress, the maximum breaking stress was further improved. These findings highlight the potential of nanofiber reinforcement in increasing the mechanical performances of composites in critical areas subjected to significant stress. Furthermore, the addition of nylon-6 (N6) to composite materials demonstrated remarkable enhancements in Young’s modulus.^26^ Specifically, the N6/YD composite exhibited a 20.5% improvement, the N6/YDJR composite showed a 49% enhancement, and the N6/JR composite displayed an extraordinary 1700% increase in Young’s modulus. Additionally, the study highlighted that incorporating N6 nanofibers critically improved the thermal stability of the epoxy resin matrices. This indicates the potential of nanofiber reinforcement in enhancing both mechanical performances and thermal performance of epoxy-based composites. Saz-orozco et al. conducted an investigation into the Mode I fracture toughness of glass fiber/vinyl ester (GF/VE) composites and explored the effects of interleaving with polyethylene terephthalate (PET) and polyamide (PA).^27^ PA nanofibers showed a better improvement compared to PET nanofibers. The propagation value of the PA-reinforced composite increased up to 90% and the initial fracture toughness value increased by 59%. In recent times, the focus of research has shifted toward electrospinning PA66 nanofibers because of their exceptional characteristics, which surpass those of other materials used in similar applications. Mechanically strong PA66 nanofibers have gained attention for their impressive attributes, including excellent manufacturability, remarkable fiber-forming capability, melting in high temperatures, and compatibility with resin. These superior properties make PA66 nanofibers an attractive choice for various applications in different forms of polyamides, leading to increased interest and exploration in the field of nanofiber reinforcement.

A large number of authors explored different uses of different PA66 solutions and reported observed advantages. In their research, Sanatgar et al. explored the electrospinning of PA66 using different solution ratios. The presence of chloroform (18% by weight) in the PA66/formic acid solution caused a reduction in solution crystals.^28^ Beckermann and Pickering studied the effects on interlayer fracture toughness, Mode I and Mode of autoclaved unidirectional (UD) carbon/epoxy composite specimens by adding polymer nanofibers interspersed in the interlayers.^29^ Aljarrah and Abdelal investigated an increase of up to 25% in the interlayer Mode I fracture toughness of carbon/epoxy laminates using different nanofiber configurations.^30^ In addition, different studies have been conducted in the literature to investigate the surface-wetting behavior of nanofibers. The polyamide-66 nanofibers were obtained to exhibit high absorbency.^31^ Studies reported that the PA66 nanofiber coating resulted in enhanced wetting, leading to a substantial decrease in the contact angle. These research findings demonstrate the potential of PA66 nanofibers in enhancing material properties and fracture toughness in various composite applications.^32,33^ Ahmadloo et al. conducted a study in which they included different PA66 solution concentrations (0.5, 1, and 3 wt %) to increase the Mode I fracture toughness of nanocomposites.^34^ The research revealed significant improvements in the maximum failure load of the material as the nanofiber cover in the epoxy increased. This suggests that the addition of PA66 nanofiber covers positively influences the fracture toughness characteristics of the epoxy-based nanocomposite. In another study by Nan Zheng et al., PA66/PCL nanofibers were used as an interlayer to increase the fracture toughness of carbon fiber/epoxy composites^35^ Saeedifar et al. observed that PA66 nanofibers reduce the toughening ability of C/E composites at high temperatures.^36^ Overall, researchers presented a wide range of advantages of electrospun PA66 nanofibers, but their use for secondary bonding is very limited. Depending on production conditions and part dimensions, secondary bonding becomes the technically optimal method in many cases. However, secondary bonding frequently suffers from adhesive and substrate bonding failure. While increasing the adhesive thickness was found to enhance the adhering of adhesive to the surface, the resulting weight gain is undesired. For such a case, secondary bonding is a very promising solution to enhance interfacial bonding.

As an extension of our previous work,^37^ the present study makes a unique contribution to a better mechanical performance understanding of electrospun PA66 nanofibers incorporation into the joint region of secondary bonded carbon fiber reinforced polymer (CFRP) composite parts. Unidirectional (UD) carbon/epoxy prepreg fabrics employed commonly in aerospace applications were used with and without PA66 nonwoven coatings to fabricate composite laminates. The nanofibers produced were directly coated onto carbon fabric. In this study, the morphology of the nanofiber layer formed by electrospinning PA66 was investigated using SEM to assess its homogeneity and absence of bead-like structures. The thermal properties of the PA66 nanofiber layer were measured using DSC. For the experiment, reference and PA66-coated composite samples were produced by using the autoclave technique. The mechanical strength of the joints was determined by various tests, including the single lap shear test, Charpy impact test, and Mode-I (DCB). Additionally, the joining region failure modes were investigated to understand how the PA66 nanofiber coating affects the joining performance. The shear strength limits of the joint were analyzed in order to measure the impact of electrospun PA66 nanofibers on the performance of the junction. These comprehensive evaluations were conducted to assess the potential enhancement in mechanical properties and toughness of the composite joints by the inclusion of electrospun PA66 nanofibers. Impact resistance and interfacial strength are observed to be improved by CRFP, which is strengthened by the PA66 nanofibers.

## 2 Experimental Section

### 2.1 Materials

UD carbon fiber/epoxy prepreg fabrics, whose unit weight is 350 g/m^2^, were employed in the study. As the adhesive, the FM300 K film adhesive was utilized. In the electrospinning process, PA66 pellets sourced from Sigma-Aldrich-429171 were used. For dissolving the PA66 pellets, formic acid (Sigma-Aldrich-27001) and chloroform (Sigma-Aldrich-24216) were selected as solvents, following the practices described in the literature.^28^

After the curing process, 0.142 mm was read as the ply thickness of the UD prepreg, while 0.16 mm was obtained as the average thickness of the FM300 K adhesive, as detailed in Table 1. The increase of the PA66 nanofiber thickness in the postcuring process was negligible and provided confirmation of an undamaged nanofiber system. Furthermore, for the initial phase, a crack along the interlaminar region of the double cantilever beam test specimens was formed by adding a polyimide film (Kapton) whose thickness was 0.05 mm at the center of the plies.

**Table 1:** Pre- and Postcure Thicknesses of UD Prepreg, FM300 K (1 Layer) Film Adhesive, Kapton Film, and PA66 Nanofibers (10 wt %)

	avg. thickness (mm)	
	pre-curing	post-curing
unidirectional prepreg	0.156	0.142
FM300 K (1 layer)	0.200	0.160
PA66 nanofibers (10 wt %)	0.021	0.020
kapton film	0,05	0,05

### 2.2 Production of PA66 Nanofibers by Electrospinning

**Figure 1:**
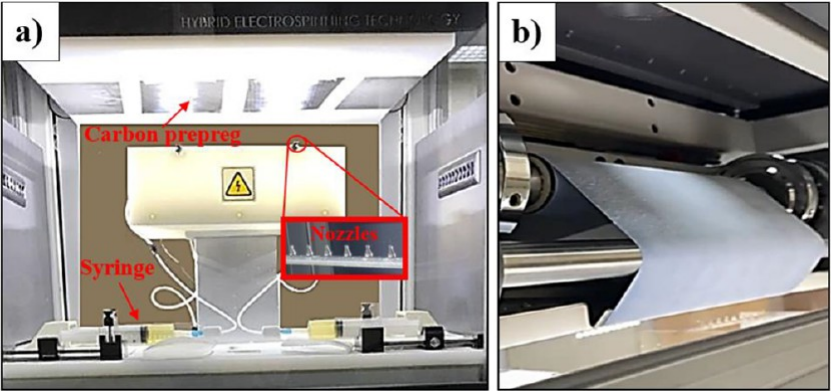
(a) Nanofiber-coated prepregs in the collector. (b) Continuous substrate winding the electro-spinning collector system with 0.01 to 5 gr/m^2^ production capacity.

Prior to preparing the solutions, PA66 pellets were subjected to a moisture removal process by heating and keeping them at 80 °C for 24 h. At room temperature, the solution ratio was established by dissolving a 10% weight ratio of PA66 pellets in 100 mL of formic acid/chloroform (75:25 v/v). The inclusion of chloroform in the PA66/formic-acid solution creates an increase in solution viscosity, promoting the production of more uniform nanofibers. The selection of this specific concentration was based on the outcomes reported in a previous study conducted by the researchers.^15^ The electrospinning device setup depicted in Figure 1 was employed for the production of PA66 nanofibers. Specifically, the researchers utilized the Innovenso PE 300 electrospinning device, which is well-suited for automation purposes, facilitating a streamlined and efficient nanofiber production process.

Two 50 mL syringes, which were filled with the PA66 polymer liquid, were connected to the propellant pump. To achieve the production of uniform and bead-free PA66 nanofibers, the researchers identified the optimal parameters through a combination of their experience and insights gleaned from relevant recommendations in the literature.^15,28^ The flow rate of the PA66 solution was set at 18 mL/h, with each nozzle operating at 1.0 mL/h. For the electrospinning process, the researchers optimized the applied voltage to be 30 kV while maintaining a nozzle-to-fiber distance of 12 cm. Thermal properties of the electrospun PA66 veils were analyzed by Differential Scanning Calorimetry (DSC). Under a nitrogen atmosphere, a spun sample extracted from the surface of the carbon underwent heating between room temperature and 350 °C, where a heating rate of 108 °C/min was performed. For the evaluation of wetting angles, by utilizing the device of the KSV Attension Theta, measured contact angles were conducted in the laboratory. Wetting angles have been obtained at three distinct locations on each surface.

### 2.3 Manufacturing of Composite Laminates

**Figure 2:**
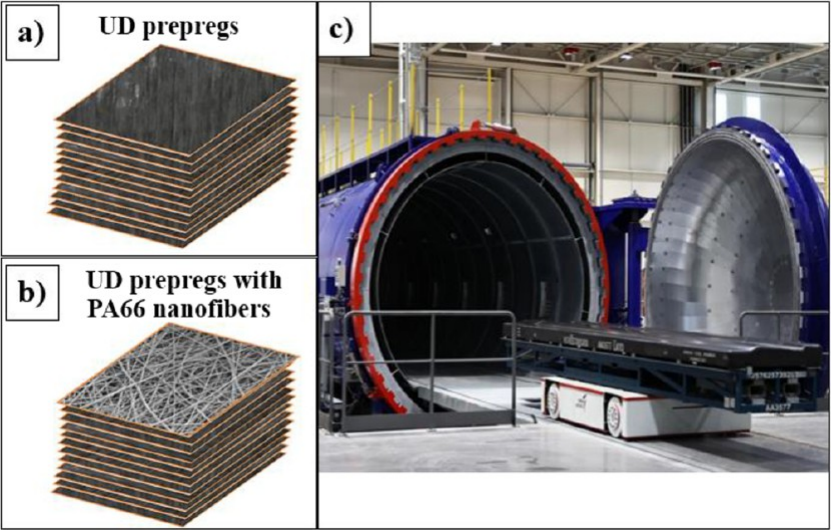
Composite laminates of 12 layers of UD prepregs at [45/0/45/90/–45/0]_s_ (a) without and (b) with electrospun PA66 nanofiber coatings. (c) Operated autoclave setup.

**Figure 3:**
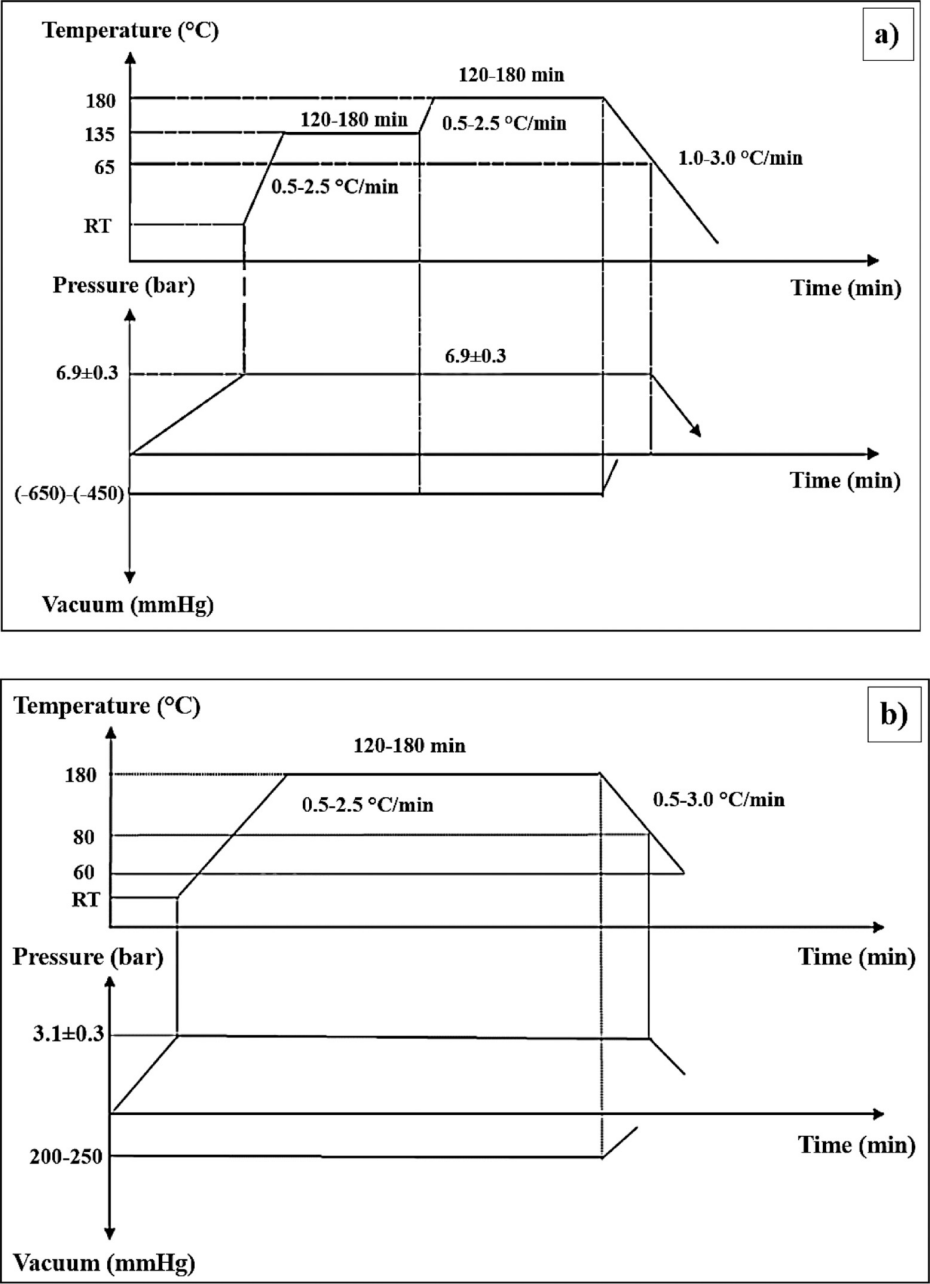
Manufacturing procedure for the autoclave curing of (a) composite laminates based on UD/UD prepregs (CFRP) and (b) composite joints using film adhesives (FM300 K).

We employed the autoclave technique to manufacture composite laminates of UD prepreg (HEXPLY - M91/IM7/34RC/UD/194/12K) CFRP at [45/–45/45/90/–45/0]_s_ order with and without electrospun PA66 nanofiber coatings (Figure 2). The fabrication procedure is listed in Figure 3.

**Figure 4:**
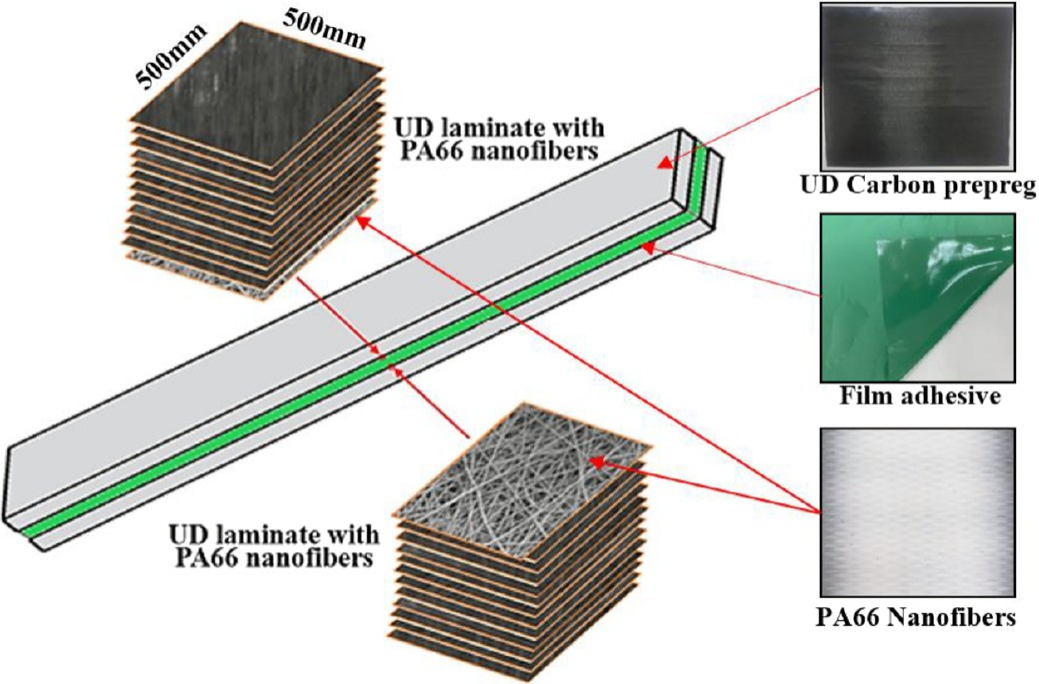
Adhesive joining of UD CFRP prepreg fabrics with the electrospun PA66 nanofibers incorporates at the joint interfaces.

PA66 nanofibers (coated for 10 min and 0.021 mm thick after coating) were added only to the first layer (joining zone) of the 12-ply prepregs (Figure 2b). The reference and PA66 nanofiber added prepregs were prepared according to the manufacturing procedure described in Figure 2a,b. Figure 2c shows the utilized autoclave setup. The prepregs were made at room temperature and placed in an autoclave. The autoclave temperature was adjusted according to the cure schedule given in Figure 3a. The prepregs were left to cure under pressure that is 7 bar and laminated finally. Before bonding, the surfaces of the fabricated laminates (composite parts), which would be bonded, were cleaned with alcohol in order to be prepared. The bonding specimens were prepared according to the manufacturing procedure described in Figure 4 by applying 3 layers of film adhesive (FM300 K) between two composite parts. The autoclave temperature was set according to the cure schedule given in Figure 3b. Bonded laminates were obtained by being left to cure under 3 bar pressure after increasing the temperature to 180 °C.

The high-viscosity FM300 K film adhesive is used to bond the UD parts together. The composite laminates with PA66 incorporated into the joint area were bonded together as described in Figure 4. These composite parts with an area of 500 × 500 m^2^ and an average thickness of 4.8 mm (12 layers of UD prepreg +3 layers of Film adhesive +12 layers of UD prepreg) were trimmed according to the test specimen dimensions described within ASTM and ISO standards.

**Figure 5:**
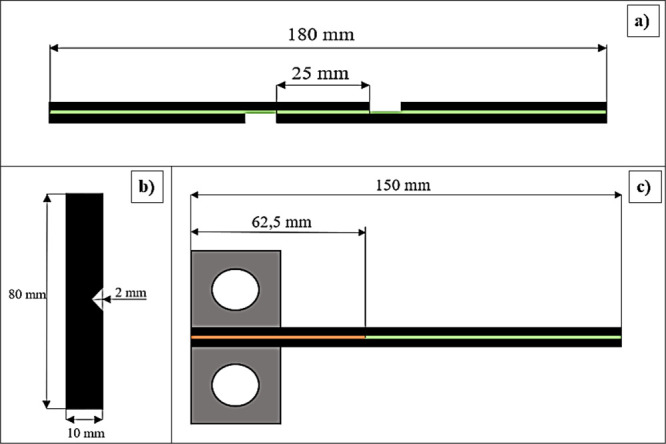
Schematic representation of (a) single lap shear, (b) Charpy, and (c) DCB test specimens.

The single lap shear test specimens are illustrated in Figure 5a,b. The DCB test specimens were 150 mm long and 25 mm wide (Figure 5c). In addition, a 62.5 mm long polyamide film (Kapton, 0.05 mm thick) was placed in the center of the plies to create an initial crack along the interlaminar region of the double cantilever beam specimens. By use of 280-grit sandpaper, the cut edges of the specimens were lightly sanded by hand.

### 2.4 Mechanical Testing

**Figure 6:**
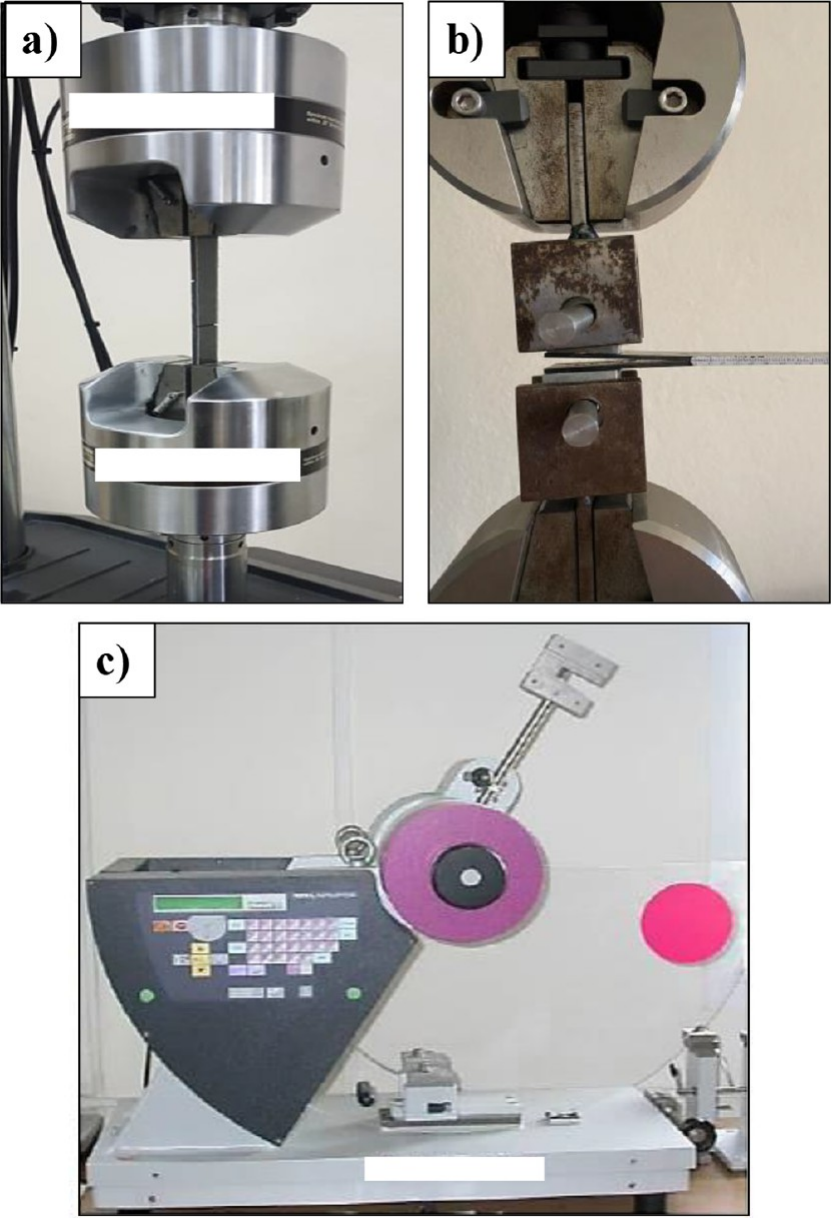
Images of the test specimens under (a) Lap shear and (b) DCB and (c) Charpy loading.

We performed Single-lap shear, Charpy impact energy, and Mode-I tests on adhesively joined unidirectional Carbon fiber composites. Figure 6a presents the configuration for the single-lap shear. The tests were performed using the MTS Landmar Servo-Hydraulic Testing System, following the guidelines specified in ASTM standard D5868.^38^Figure 6c presents the Charpy testing setup. The CEAST Resil Impactor was used for Charpy impact tests (max. Fifteen J - 25 J). The specimens were manufactured in accordance with the ISO-179 standard.^39^ Charpy impact strength is determined by the ratio of the energy absorbed during the impact test to the notched area of the sample.

The Mode-I laminar fracture toughness of the composite specimens was evaluated by using the DCB test. The tests were performed on a Shimadzu AGS-X instrument using the ASTM D5528 test standard. The configuration of the DCB test specimens is illustrated in Figure 6b. The test results were recorded and the Mode I interlayer fracture toughness (*G*_IC_) value was calculated using the ASTM standard and the Modified Beam Theory data reduction method.^33,40,41^

## 3 Results and Discussion

**Figure 7:**
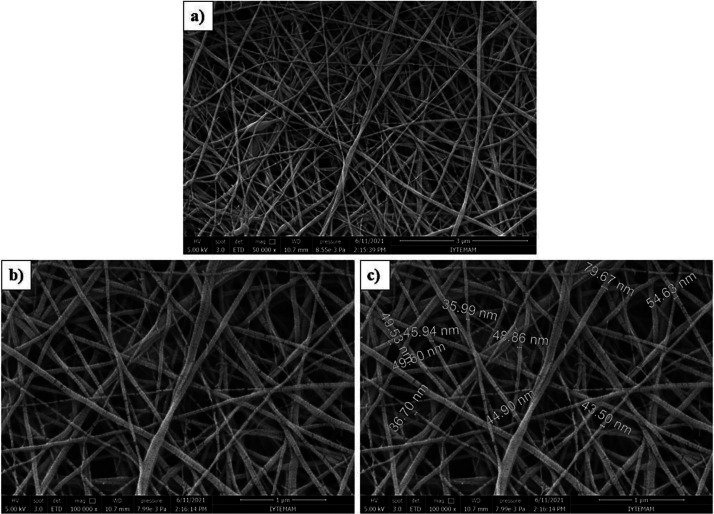
SEM images of 10% by weight PA66 nanofibers at magnification of (a) 50,000×, (b) 100,000×, and (c) 100,000×.

Based on the procedure described, we obtain a uniform coating of electrospun nanofibers onto the surface of carbon prepregs. After electrospinning, nanofiber deposition was observed as a color change of the prepreg surfaces (Figure 1b). The resulting nanofiber diameters are given through the SEM image in Figure 7c. The produced nanofibers create a beadless mesh network that is uniform and continuous. Nanofiber diameters were measured by taking 10 different measurements from nanofibers. Minimum, maximum, and average nanofiber diameters were calculated as 35.99, 79.67, and 48.96 nm, respectively.

**Figure 8:**
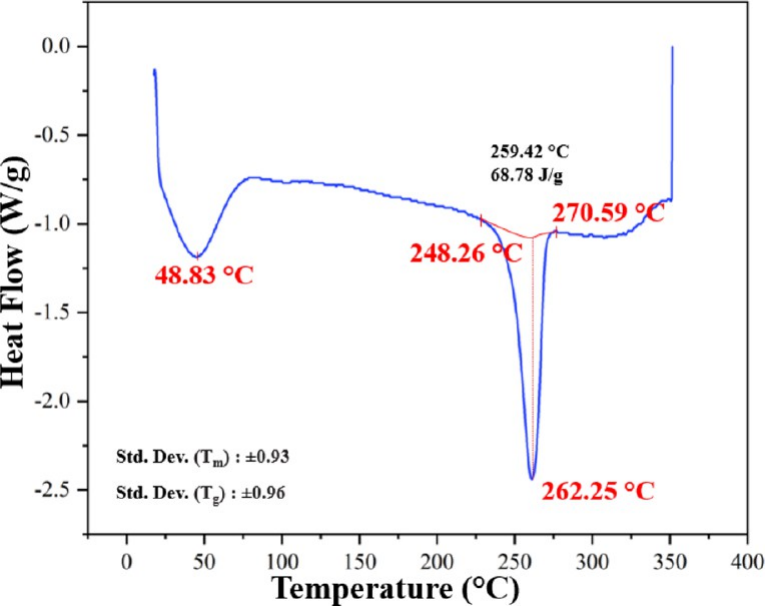
DSC curve of PA66 veils.

The DSC curves of the PA66 nanofibers are given in Figure 8. For these nanofibers, 262.25 and 48.83 °C were the measurements for melting temperature (*T*_m_) and the glass transition temperature (*T*_g_), respectively.

**Figure 9:**
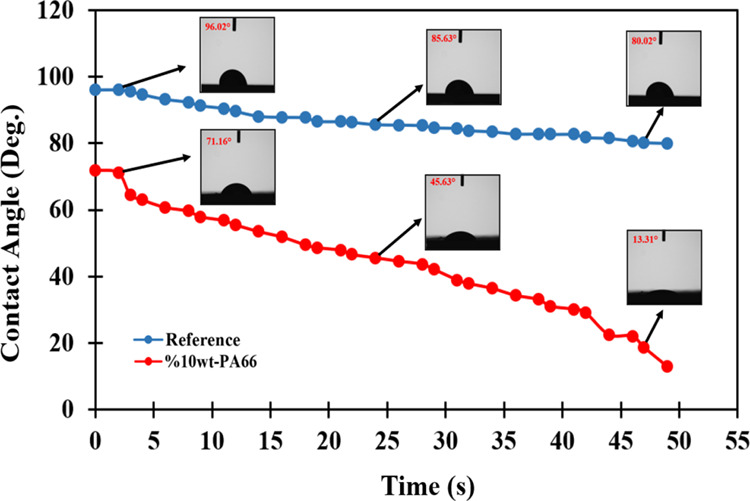
Droplet images and wetting angle variations of 10 wt %-PA66 coated surface and uncoated prepreg surface (reference) by time.

Figure 9 illustrates the difference in contact angles on both bare and PA66 nanofiber-coated surfaces. On the bare prepreg surface, the water droplet retains a spherical bead form, with only a slight decrease in the contact angle that is caused by the reduced water absorption of the composite system. The contact angle observed on uncoated prepreg surfaces approaches hydrophobic behavior, measuring around 80°. This indicates that the uncoated prepreg surface exhibits characteristics similar to hydrophobic materials, which tend to repel water and form relatively high contact angles with water droplets. In contrast, very strong water absorption was observed to be developed in the case of surface coated by PA66 nanofibers.

**Figure 10:**
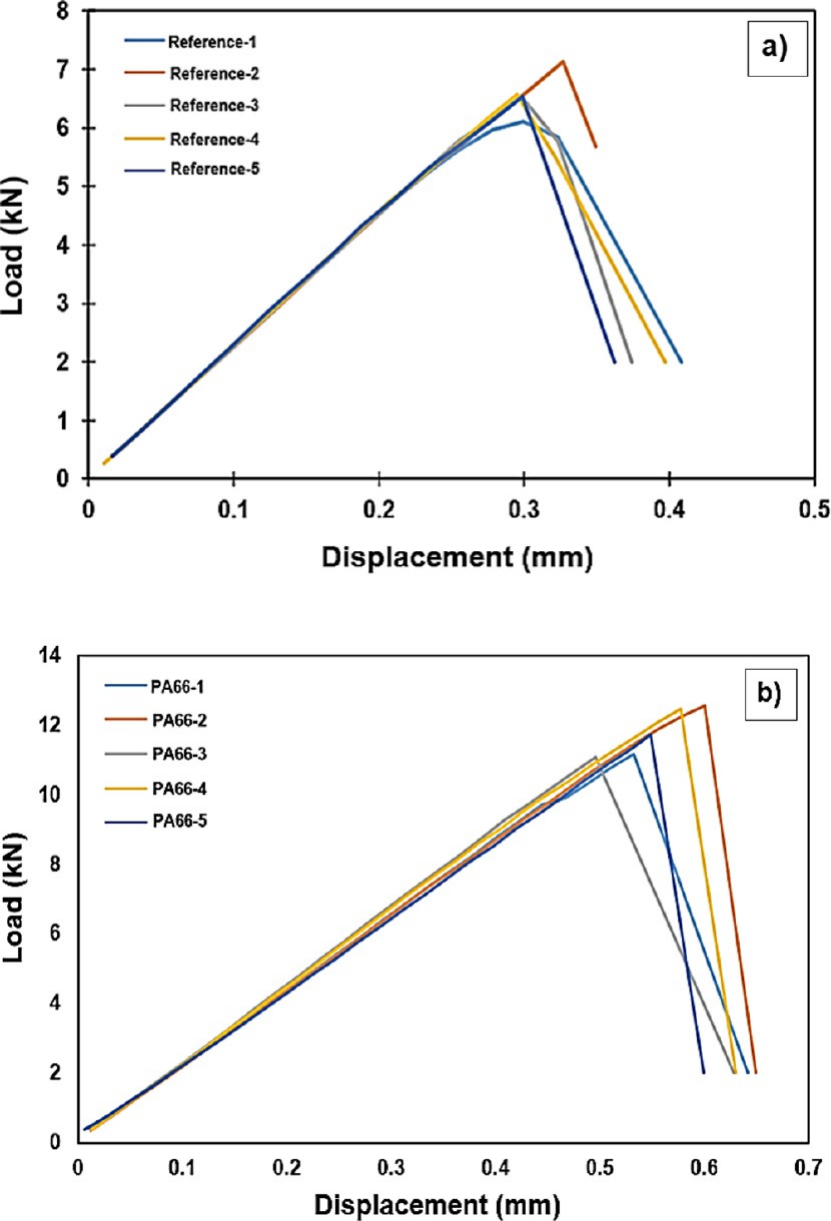
Load vs displacement curves of single lap shear tests of five different samples of (a) uncoated reference and (b) 10 wt %-PA66-3 coated surfaces joined using 3 FM300 K plies.

We performed lap shear tests according to ASTM D5868 as the load–displacement curves are given in Figure 10. A linear load–displacement behavior is observed at the first stage of loading while the max. shear strengths of the reference and PA66 added composites produced are 7.13 and 12.55 kN, respectively. During the test, the same amount of preload was applied to each specimen to prevent any backlash after the specimens were connected to the jaws of the device.

**Figure 11:**
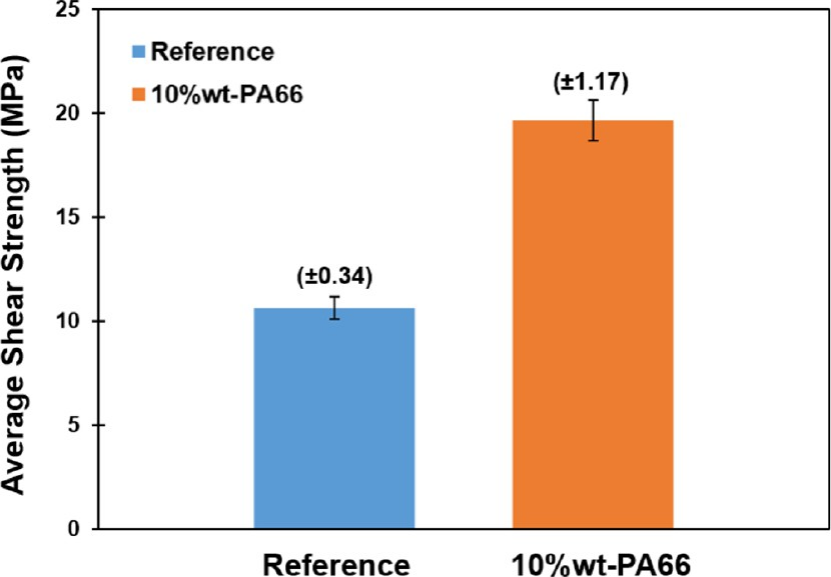
Average values for lap shear strength of test specimens.

Figure 11 provides a summary of the results of the lap shear tests conducted on composite specimens with PA66 (10 wt %) added to both attachment regions and the reference regions. Before reaching the point of fracture, both the reference and the PA66 nanofiber-reinforced samples exhibit linear elastic behavior. Comparison between PA66 nanofiber-reinforced samples and the reference sample indicates that PA66 nanofiber-reinforced samples have better performance up to 78.63%. This advancement in shear strength highlights the positive effect of incorporating PA66 nanofibers into the composite material, leading to enhanced mechanical properties and improved performance in lap shear tests. The 78.63% improvement indicates the efficacy of the PA66 nanofiber addition in reinforcing the composite, which is beneficial in various applications requiring increased strength and durability.

**Figure 12:**
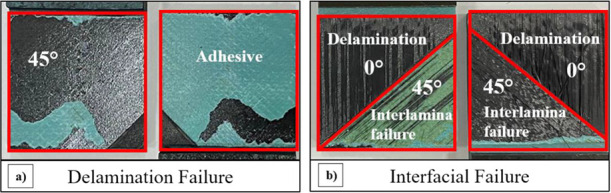
Fracture surface of (a) reference samples and (b) 10 wt % PA66 specimens after a single lap shear test.

Figure 12 examines the fractured surfaces in the joint area of PA66 uncoated and PA66 coated composites after SLS testing. Interfacial debonding is the only failure mode in the reference samples (Figure 12a). This is associated with the lowest strength of the adhesive. In the case of the PA66-added specimens, as shown in Figure 12b, the fractured surfaces exhibit some differences when compared with the reference specimens. Notably, the failure model exhibited shows that 45-degree layers cover half of the damaged surface. On the other hand, when the other half of the fracture surface is examined, delamination is observed between both 0-degree and 45-degree layers. When the fracture surfaces of the PA66-added and nonadded samples were examined, it was determined that the PA66-added samples adhered more strongly to the adhesive compared to the nonadded samples.

When the fracture surface images and the test results were examined, it was proven that adding PA66 nanofibers to the joint region caused a strong improvement in the strength of the joint region. This structure, prepared by adding PA66, was identified as an excellent method to increase strength by reducing the fragility of the joining zone.

**Figure 13:**
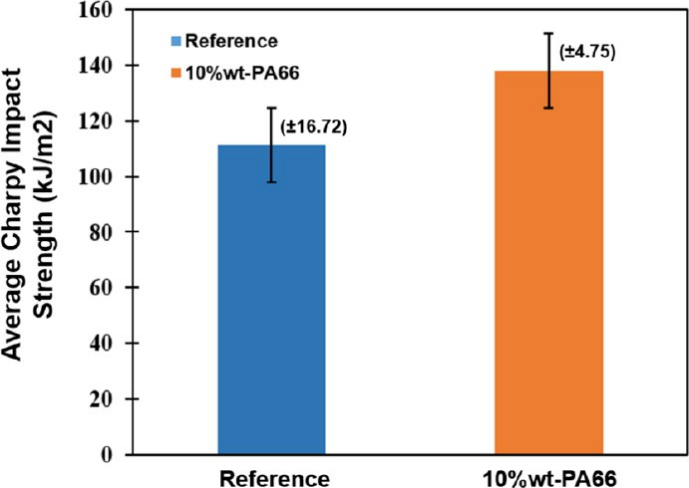
Charpy impact energy of composite samples with/without PA66 nanofiber.

**Figure 14:**
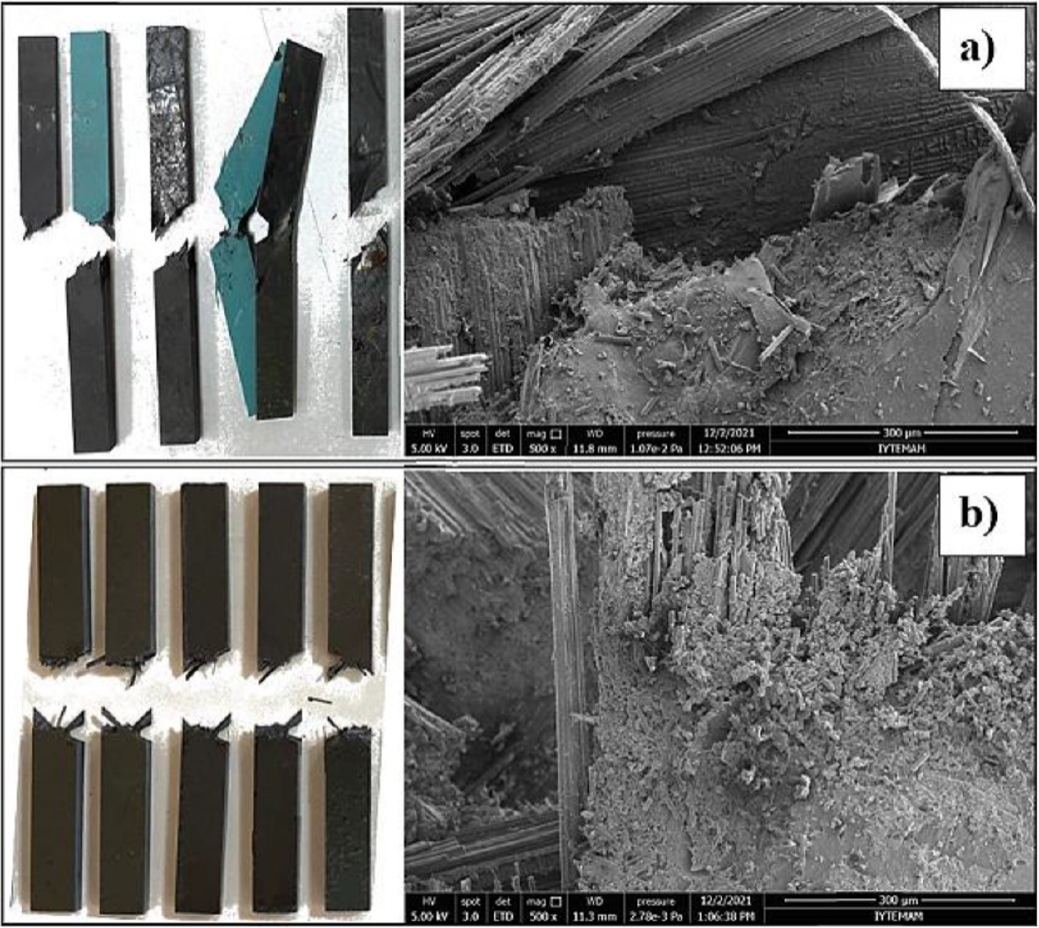
Image of (a) bare and (b) PA66 added specimens after the Charpy test and the joint region SEM images.

The Charpy impact energies of the reference and PA66-added samples were 111.2 and 137.8 kJ/m^2^, respectively (Figure 13). When the composites were modified with PA66, the charpy impact energy increased by approximately 24%. After the test, specimen images and SEM results were analyzed (Figure 14). The findings from the study reveal that in comparison to the reference specimens, the PA66 nanofiber interspersed specimens demonstrated a fracture surface in the epoxy matrix that was more complex and irregular. This indicates that the addition of PA66 nanofibers led to higher plastic deformation and increased energy absorption during impact events. The improvement observed in the PA66 nanofiber-reinforced specimens can be attributed to the presence of nanofibers within the junction regions. These nanofibers contributed significantly to enhancing the cracking resistance during impact, thereby increasing the load-absorbing capacity of the specimens and their overall resistance to failure damage. The PA66 nanofibers were effective energy absorbers for the composite material. It dissipated the impact energy and prevented catastrophic failures. This behavior highlights the advantageous role of PA66 nanofibers in enhancing the impact resistance and mechanical performance of the composite material.

**Figure 15:**
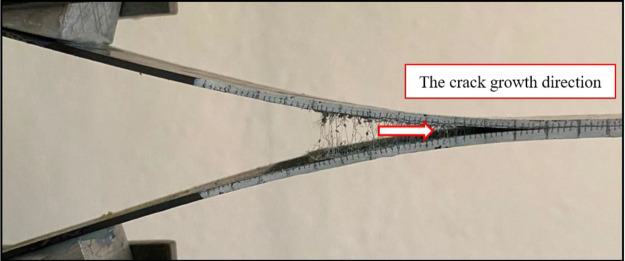
Photographs of PA66 composite specimens under Mode-I loading.

Based on the Mode I test results, Fmax data for the reference composite specimens and the PA66 added composite specimens were determined as 64.5 and 79.1 N, respectively. During the testing process, PA66 nanofibers played a vital role in resisting crack propagation, consequently increasing the Mode I value of the composite material (Figure 15). The nanofibers acted as an effective bonding agent, firmly holding the carbon layers and adhesive together. This interfacial reinforcement provided by the PA66 nanofibers significantly resisted crack propagation and resulted in enhanced energy absorption capabilities within the composites.

**Figure 16:**
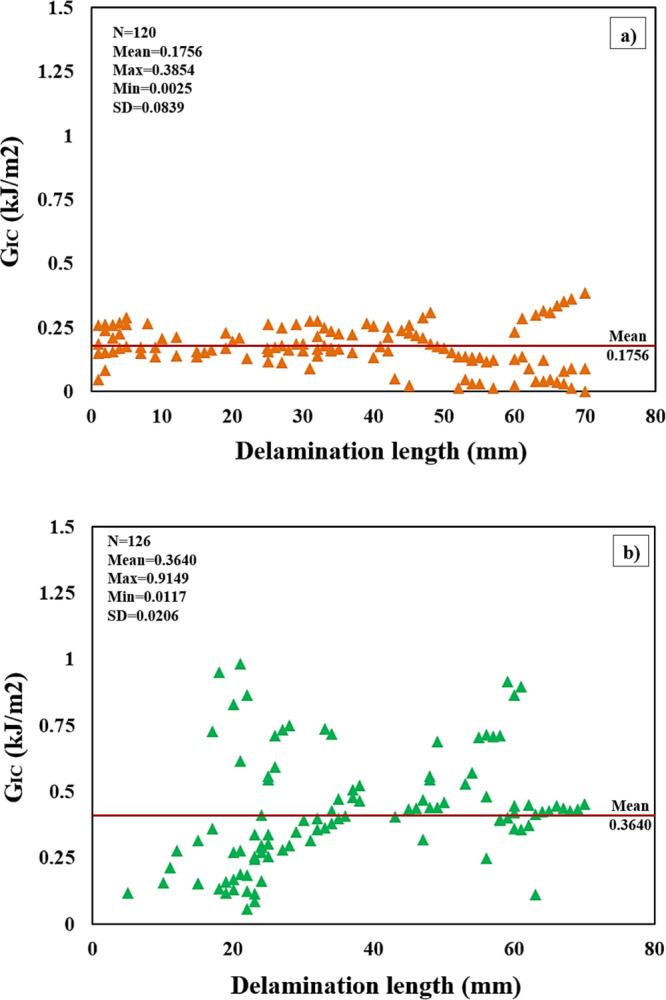
*G*_IC_ and delamination length curve of (a) PA66 nonadded and (b) PA66 added composite samples.

The *G*_IC_ and delamination length curves of PA66 unmodified and PA66 modified test specimens are shown in Figure 16. The average *G*_IC_ value of the PA66 unmodified test specimens was calculated as 0.1756 kJ/m^2^. The average *G*_IC_ value of PA66-added composites was calculated as 0.3640 kJ/m^2^. When the samples with and without PA66 were compared, it was observed that the Mod-I value was improved by approximately 107%. The *G*_IC_ and delamination length curves for both reference and PA66 added composite specimens are presented in Figure 16. The mean *G*_IC_ value of the nonadded composites was calculated to be 0.1756 kJ/m^2^. In contrast, the mean *G*_IC_ value of the PA66-added composites was determined to be 0.3640 kJ/m^2^. By comparing the PA66-added composite samples with the nonadded composite samples, it becomes evident that the Mode-I value was significantly improved by approximately 107%. This substantial enhancement in fracture toughness demonstrates the positive impact of incorporating PA66 nanofibers into the composite material. The PA66 nanofibers effectively contributed to strengthening the interlaminar bonding and increasing the resistance to crack propagation, leading to a significant improvement in the composite’s ability to withstand Mode-I loading conditions. The result indicates that the addition of PA66 nanofibers has notably enhanced the overall mechanical performance and fracture resistance of the composite material.

**Figure 17:**
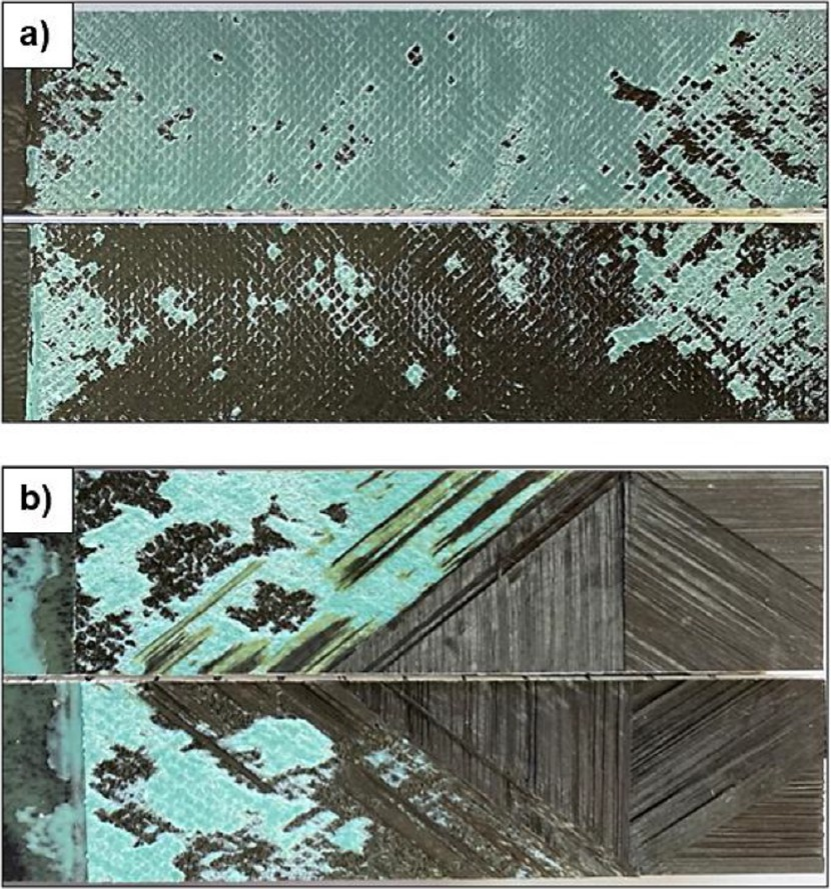
Photograph of the DCB surfaces of fractured (a) reference and (b) PA66 added composite.

The fracture surfaces of the nonadded reference and PA66 added samples after the double cantilever beam testing can be seen in Figure 17. Notably, the PA66-coated composite samples exhibited a distinct fracture behavior compared to the reference samples. In the nonmodified samples, the separation occurred primarily in the adhesion region. However, in the modified samples, initiation of separation propagated to the lamina side after occurring at the adhesive side. The presence of PA66 nanofibers on the composite surface increased the surface area and roughness, leading to enhanced bonding for the composite surface and adhesive surface. As a result, the strength of the bonding area was importantly improved. The incorporation of electrospun nanofibers transformed the adhesive failure mode from cohesive adhesion failure to interlaminar failure. This transformation results in an essential improvement in the adhesion performance of the joints. The nanofibers effectively acted as a reinforcing layer, enhancing the interfacial adhesion between the composite substrates and adhesive layers. Consequently, the joints containing nanofiber-coated composite substrates achieved relatively higher bond strength values compared to the reference specimens without nanofiber coatings. The nanofiber layers on the surface of the composite were major drivers in finding out the failure mode and providing improved bond strength. In summary, the incorporation of nanofiber layers on the composite surface was instrumental in achieving greater interfacial adhesion values between the composite substrates and adhesive layers. This, in turn, led to enhanced bond strength and improved adhesion performance in the composite joints. The electrospun nanofibers were crucial for transforming the adhesive failure mode and contributing to the overall mechanical performance of the composite joints.

## 4 Concluding Remarks

In order to develop the mechanical capabilities of CF/EP composites, PA66 nanofibers were incorporated into the bonding region of composite joints as a content of this study. To determine the impact of PA66 interleaving systems on the mechanical performance of CF/EP composites, some mechanical test studies were performed. It can be read from results that strength in the Single lap shear test and Charpy impact test are improved by approximately 79% and 24%, respectively, by incorporation of the PA66 nanofibers into the joint region. The results also showed that by using PA66 nanofibers, the Mode-I fracture toughness value was increased by approximately 107%. By combining PA66 nanofibers with the joint regions of the composites, some properties of the composites were enhanced, such as impact damage energy absorption, shear strength, and fracture toughness.

## References

[ref1] Noor A. K.; Venneri S. L.; Paul D. B.; Hopkins M. A. Structures technology for future aerospace systems. Comput. Struct. 2000, 74 (5), 507–519. 10.1016/S0045-7949(99)00067-X.

[ref2] Encinas N.; Oakley B. R.; Belcher M. A.; Blohowiak K. Y.; Dillingham R. G.; Abenojar J.; Martínez M. A. Surface modification of aircraft used composites for adhesive bonding. Int. J. Adhes. Adhes. 2014, 50, 157–163. 10.1016/j.ijadhadh.2014.01.004.

[ref3] Brito C. B. G.; De Cássia Mendonca Sales Contini R.; Gouvêa R. F.; De Oliveira A. S.; Arbelo M. A.; Donadon M. V. Mode I interlaminar fracture toughness analysis of Co-bonded and secondary bonded carbon fiber reinforced composites joints. Mater. Res. 2017, 20, 873–882. 10.1590/1980-5373-mr-2016-0805.

[ref4] Shin K. C.; Lee J. J.; Lee D. G. A study on the lap shear strength of a co-cured single lap joint. J. Adhes. Sci. Technol. 2000, 14 (1), 123–139. 10.1163/156856100742140.

[ref5] Omairey S.; Jayasree N.; Kazilas M. Defects and uncertainties of adhesively bonded composite joints. SN Appl. Sci. 2021, 3 (9), 1–14. 10.1007/s42452-021-04753-8.

[ref6] Ke L.; Li C.; Luo N.; He J.; Jiao Y.; Liu Y. Enhanced comprehensive performance of bonding interface between CFRP and steel by a novel film adhesive. Compos. Struct. 2019, 229, 111393 10.1016/j.compstruct.2019.111393.

[ref7] Wang C. H.; Chalkley P. Plastic yielding of a film adhesive under multiaxial stresses. Int. J. Adhes. Adhes. 2000, 20 (2), 155–164. 10.1016/S0143-7496(99)00033-0.

[ref8] Song M. G.; Kweon J. H.; Choi J. H.; Byun J. H.; Song M. H.; Shin S. J.; Lee T. J. Effect of manufacturing methods on the shear strength of composite single-lap bonded joints. Compos. Struct. 2010, 92 (9), 2194–2202. 10.1016/j.compstruct.2009.08.041.

[ref9] Moretti L.; Olivier P.; Castanié B.; Bernhart G. Experimental study and in-situ FBG monitoring of process-induced strains during autoclave co-curing, co-bonding and secondary bonding of composite laminates. Composites, Part A 2020, 142, 106224 10.1016/j.compositesa.2020.106224.

[ref10] Mohan J.; Ivanković A.; Murphy N. Mode i fracture toughness of co-cured and secondary bonded composite joints. Int. J. Adhes. Adhes. 2014, 51, 13–22. 10.1016/j.ijadhadh.2014.02.008.

[ref11] Li X.; Tao R.; Yudhanto A.; Lubineau G. How the spatial correlation in adhesion properties influences the performance of secondary bonding of laminated composites. Int. J. Solids Struct. 2020, 196–197, 41–52. 10.1016/j.ijsolstr.2020.04.012.

[ref12] Budhe S.; Banea M. D.; De Barros S.; Da Silva L. F. M. An updated review of adhesively bonded joints in composite materials. Int. J. Adhes. Adhes 2017, 72, 30–42. 10.1016/j.ijadhadh.2016.10.010.

[ref13] Mohan J.; Ivanković A.; Murphy N. Mixed-mode fracture toughness of co-cured and secondary bonded composite joints. Eng. Fract. Mech. 2015, 134, 148–167. 10.1016/j.engfracmech.2014.12.005.

[ref14] Mazumdar S. K.; Mallick P. K. Static and fatigue behavior of adhesive joints in SMC-SMC composites. Polym. Compos. 1998, 19 (2), 139–146. 10.1002/pc.10084.

[ref15] Beylergil B.; Tanoğlu M.; Aktaş E. Enhancement of interlaminar fracture toughness of carbon fiber–epoxy composites using polyamide-6,6 electrospun nanofibers. J. Appl. Polym. Sci. 2017, 134 (35), 45244 10.1002/app.45244.

[ref16] Mohan A. Formation and Characterization of Electrospun Nonwoven Webs, Text. Manag. Technol., 2002, [Online]. Available: https://www.mendeley.com/viewer/?fileId=789cf426-0a97-dd89-4e13-fa027cfe584a&documentId=c2902bde-2e32-39df-80cd-8f3b76838cea.

[ref17] Lyons J.; Li C.; Ko F. Melt-electrospinning part I: Processing parameters and geometric properties. Polymer 2004, 45 (22), 7597–7603. 10.1016/j.polymer.2004.08.071.

[ref18] Kim C.; Park S. H.; Lee W. J.; Yang K. S. Characteristics of supercapaitor electrodes of PBI-based carbon nanofiber web prepared by electrospinning. Electrochim. Acta 2004, 50 (2–3), 877–881. 10.1016/j.electacta.2004.02.071.

[ref19] LeLam H. Electrospinning of Single Wall Carbon Nanotube Reinforced Aligned Fibrils and Yarns. A Thesis Submitt. to Fac. Drexel Univ., vol 2004, 2004; p 246 [Online]. Available:http://onlinelibrary.wiley.com/doi/10.1002/cbdv.200490137/abstract.

[ref20] Niu H.; Wang X.; Lin T. Needleless electrospinning: Influences of fibre generator geometry. J. Text. Inst. 2012, 103 (7), 787–794. 10.1080/00405000.2011.608498.

[ref21] Jentzsch E.; Gül Ö.; Öznergiz E. A comprehensive electric field analysis of a multifunctional electrospinning platform. J. Electrostat. 2013, 71 (3), 294–298. 10.1016/j.elstat.2012.12.007.

[ref22] Retolaza A.; Eguiazábal J. I.; Nazábal J. Structure and mechanical properties of polyamide-6,6/poly(ethylene terephthalate) blends. Polym. Eng. Sci. 2004, 44 (8), 1405–1413. 10.1002/pen.20136.

[ref23] Van der Heijden S.; et al. Interlaminar toughening of resin transfer moulded glass fibre epoxy laminates by polycaprolactone electrospun nanofibres. Compos. Sci. Technol. 2014, 104, 66–73. 10.1016/j.compscitech.2014.09.005.

[ref24] Herwan J.; Al-Bahkali E.; Khalil K. A.; Souli M. Load bearing enhancement of pin joined composite laminates using electrospun polyacrylonitrile nanofiber mats. Arab. J. Chem. 2016, 9 (2), 262–268. 10.1016/j.arabjc.2015.03.019.

[ref25] Bilge K.; Venkataraman S.; Menceloglu Y. Z.; Papila M. Global and local nanofibrous interlayer toughened composites for higher in-plane strength. Composites, Part A 2014, 58, 73–76. 10.1016/j.compositesa.2013.12.001.

[ref26] Gavande V.; Nagappan S.; Seo B.; Cho Y. S.; Lee W. K. Transparent nylon 6 nanofibers-reinforced epoxy matrix composites with superior mechanical and thermal properties. Polym. Test. 2023, 122, 108002 10.1016/j.polymertesting.2023.108002.

[ref27] Saz-orozco D.; Ray D.; Stanley W. F. Effect of Thermoplastic Veils on Interlaminar Fracture Toughness of a Glass Fiber/Vinyl Ester Composite, 2015.

[ref28] Sanatgar R. H.; Borhani S.; Ravandi S. A. H.; Gharehaghaji A. A. The influence of solvent type and polymer concentration on the physical properties of solid state polymerized PA66 nanofiber yarn. J. Appl. Polym. Sci. 2012, 126 (3), 1112–1120. 10.1002/app.36871.

[ref29] Beckermann G. W.; Pickering K. L. Mode i and Mode II interlaminar fracture toughness of composite laminates interleaved with electrospun nanofibre veils. Composites, Part A 2015, 72, 11–21. 10.1016/j.compositesa.2015.01.028.

[ref30] Aljarrah M. T.; Abdelal N. R. Improvement of the mode I interlaminar fracture toughness of carbon fiber composite reinforced with electrospun nylon nanofiber. Composites, Part B 2019, 165, 379–385. 10.1016/j.compositesb.2019.01.065.

[ref31] Kang D. H.; Kang H. W. Surface energy characteristics of zeolite embedded PVDF nanofiber films with electrospinning process. Appl. Surf. Sci. 2016, 387, 82–88. 10.1016/j.apsusc.2016.06.096.

[ref32] Stachewicz U.; Barber A. H. Enhanced wetting behavior at electrospun polyamide nanofiber surfaces. Langmuir 2011, 27 (6), 3024–3029. 10.1021/la1046645. 21332217

[ref33] Beigmoradi R.; Samimi A.; Mohebbi-Kalhori D. Fabrication of polymeric nanofibrous mats with controllable structure and enhanced wetting behavior using one-step electrospinning. Polymer 2018, 143, 271–280. 10.1016/j.polymer.2018.04.025.

[ref34] Ahmadloo E.; Gharehaghaji A. A.; Latifi M.; Mohammadi N.; Saghafi H. How fracture toughness of epoxy-based nanocomposite is affected by PA66 electrospun nanofiber yarn. Eng. Fract. Mech. 2017, 182, 62–73. 10.1016/j.engfracmech.2017.07.011.

[ref35] Zheng N.; Liu H. Y.; Gao J.; Mai Y. W. Synergetic improvement of interlaminar fracture energy in carbon fiber/epoxy composites with nylon nanofiber/polycaprolactone blend interleaves. Composites, Part B 2019, 171, 320–328. 10.1016/j.compositesb.2019.05.004.

[ref36] Saeedifar M.; Saghafi H.; Mohammadi R.; Zarouchas D. Temperature dependency of the toughening capability of electrospun PA66 nanofibers for carbon/epoxy laminates. Compos. Sci. Technol. 2021, 216, 109061 10.1016/j.compscitech.2021.109061.

[ref37] Esenoğlu G.; Barisik M.; Tanoğlu M.; Yeke M.; TÚrkdoğan C.; İplikçi H.; Martin S.; Nuhoğlu K.; Aktaş E.; Dehneliler S.; İriş M. E. Improving adhesive behavior of fiber reinforced composites by incorporating electrospun Polyamide-6, 6 nanofibers in joining region. J. Compos. Mater. 2022, 56 (29), 4449–4459. 10.1177/00219983221133478.

[ref38] (D5868) Standard Test Method for Lap Shear Adhesion for Fiber Reinforced Plastic (FRP) Bonding1 ASTM, ASTM D5868_01.pdf, *Stand. Test Method L. Shear Adhes. Fiber Reinf. Plast. Bond.,* 2001; p2

[ref39] ISO 179-1 Plastics - Determination of Charpy impact properties. ISO - Int. Stand Organ., 2010, vol 1110; pp 1–11

[ref40] Nurashikin S.; Hazizan A. J. Compos. Mater. 2011, 46 (2), 183–191. 10.1106/002199802026980.

[ref41] Standard test method for mode I interlaminar fracture toughness of unidirectional fiber-reinforced polymer matrix composites. Am. Stand. Test. Methods 2014, 03, 1–12. 10.1520/D5528-13.2.

